# Urinary chemistry in healthy cross-bred pet rabbits (*Oryctolagus cuniculus*) and rabbits with suspected chronic kidney disease

**DOI:** 10.1093/tas/txaf002

**Published:** 2025-01-29

**Authors:** Yeh Sze-Yu, Sung Chi-Hsuan, Liu Pin-Chen, Wu Ching-Fen, Lin Tsai-Lu, Cheng Tsung-Li, Chi-Chung Chou

**Affiliations:** Department of Veterinary Medicine, College of Veterinary Medicine, National Chung-Hsing University, Taichung 40227, Taiwan; Department of Veterinary Medicine, College of Veterinary Medicine, National Chung-Hsing University, Taichung 40227, Taiwan; Gastrointestinal Lab, Department of Small Animal Clinical Sciences, College of Veterinary Medicine, Texas A&M University, College Station, TX 77843, USA; Department of Veterinary Medicine, College of Veterinary Medicine, National Chung-Hsing University, Taichung 40227, Taiwan; Department of Veterinary Medicine, College of Veterinary Medicine, National Chiayi University, Chiayi City, Taiwan; Department of Veterinary Medicine, College of Veterinary Medicine, National Chung-Hsing University, Taichung 40227, Taiwan; Animal and Plant Health Inspection Agency, Ministry of Agriculture, Taipei City 100060, Taiwan; Veterinary Medical Teaching Hospital, College of Veterinary Medicine, National Chung-Hsing University, Taichung 40227, Taiwan; Department of Veterinary Medicine, College of Veterinary Medicine, National Chung-Hsing University, Taichung 40227, Taiwan; Department and Graduate Institute of Pharmacology, National Defense Medical Center, Taipei 11490, Taiwan

**Keywords:** kidney disease, *Oryctolagus cuniculus*, rabbit, reference intervals, urinary biomarkers

## Abstract

Renal biomarkers for early detection of decreased kidney function have been extensively studied in dogs and cats, but there is limited research for pet rabbits. Specifically, studies on urinary indices for cross-bred rabbits are scarce. Therefore, this study aimed to assess the potential use of urinalysis in cross-bred pet rabbits. Urine samples from 30 healthy crossbred pet rabbits and 11 rabbits with suspected kidney disease (KD) from three clinics were collected. RIs of urinary indices, including urinary protein, creatinine (uCRE), urinary protein-to-creatinine (UPC) ratio, gamma-glutamyl transferase (uGGT), and uGGT ratio, were established and compared to two published reference intervals (RIs). The results showed that healthy rabbits consistently had low urine protein levels and abnormal UPC ratios, compared to a published study involving only pure-bred rabbits. Rabbits with KD had higher urine protein levels and a significantly higher UPC ratio (*P* < 0.001), with 63% having ratios greater than 0.4. Additionally, rabbits with KD showed significantly (*P* < 0.001) lower uCRE levels and urine specific gravity (USG) with elevated uGGT index (*P* < 0.05), compared to the healthy group. Significant differences (*P* < 0.05) were also observed in urine color, turbidity, pH, and positivity of the occult blood by dipstick. This study underscores potential breed-specific variations in urinary protein levels and UPC ratio, as well as highlights the diagnostic potential of USG, UPC ratio, and uGGT index in rabbits with KD. However, the presence of breed-specific variations and technical nuances in laboratory equipment necessitate careful interpretation of results. Therefore, further studies across larger and more diverse rabbit populations are crucial to validate the diagnostic performance of urinary indices in diagnosing KD in rabbits.

## Teaser

The study underscores potential cross-breed specific variations in urinary protein levels and UPC ratio and highlights the diagnostic potential of USG, UPC ratio, and uGGT index in rabbits with kidney disease.

## Introduction

Kidney disease (KD) in rabbits is a significant health concern, particularly in older subjects. The epidemiology of KD in rabbits is not as well-documented as in other companion animals, but available studies indicate that it is a common condition (Reavill and Lennox, 2020; Shiga et al., 2022). Chronic KD in pet rabbits is often due to chronic interstitial nephritis, which can be associated with bacterial infections, viral infections, or parasitic infestations such as *Encephalitozoon cuniculi* (Reusch et al., 2009). Other contributing factors include dietary imbalances, genetic predispositions, and environmental stressors. Clinical signs of KD in rabbits can be subtle and may include weight loss, lethargy, increased water intake, and changes in urination patterns. Early detection and management are crucial to improving the quality of life and longevity of affected rabbits.

Diagnostic serum and urine biochemistry for KDs have been widely studied in dogs and cats, but studies on urinary indices for KD in cross-bred pet rabbits are limited (Kraus et al., 1984). Currently, creatinine (CRE) is the primary diagnostic tool for the preliminary identification and evaluation of KD severity in rabbits; however, its sensitivity is relatively low. Additional renal biomarkers for early detection of decreased kidney function and damage localization are desirable. Urinalysis biomarkers, such as urine protein to creatinine (UPC) ratio, urine specific gravity (USG), and urinary γ-glutamyl transferase to creatinine ratio (uGGT index), are well-studied in dogs and cats. Due to the noninvasive nature of voided urinary samples, urinary biochemistry may be useful for assessing renal function in rabbits.

Normally, young rabbits have small amounts of albumin (Kraus et al., 1984), while adult healthy rabbits have been traced to small amounts of proteinuria from the mucus-secreting glands in the renal pelvis (Percy and Barthold, 2013). To the authors’ knowledge, there are few research reports on the association between UPC ratio and KD in rabbits. Reusch et al. (2009) established the reference interval (RI) of UPC ratio to be from 0.11 to 0.40 in the healthy rabbit population and showed that interstitial nephritis caused by *Encephalitozoon cuniculi* infection was not associated with the change of UPC ratio. In agreement with this RI, Selleri et al. (2015) found that 85% of rabbits with KD have a UPC ratio greater than 0.4. Unlike cats and dogs, rabbits have a limited ability to concentrate urine. The reported normal range of USG for rabbits was from 1.003 to 1.036 (Meredith and Flecknell, 2006) or 1.010 to 1.057 in the healthy rabbit population (Reusch et al., 2009), without the knowledge of the range of isosthenuria. The USG could help differentiate prerenal from renal azotemia and detect compromised concentrating ability due to tubular damage. To access renal tubular damage, urinary γ-glutamyl transferase (uGGT), typically located within the proximal tubular cells, can be released into the lumen or urine when tubular injury occurs. The uGGT is used to detect early acute renal tubular injury and nephrotoxicity. Mancinelli et al established the reference intervals (RIs) of uGGT to be 2.7 to 96.5 IU/L and the uGGT index to be 0.043 to 1.034 in the healthy rabbit population (Mancinelli et al., 2012). Given the lack of literature on routine urinary parameters for KDs in rabbits, this study aimed to establish RIs of urinary indices (USG, UPC ratio, and uGGT index) and explore the potential application of urinary indices in diagnosing KD in crossbred pet rabbits.

## Methods

### Case Selection

The study protocol was reviewed and approved by the Institutional Animal Care and Use Committee of National Chung Hsing University (IACUC approval No.: 107–010). Both inpatients and outpatients were collected from client-owned rabbits that attended to National Taiwan University Veterinary Teaching Animal Hospital, Darwin Animal Hospital, or National Animal Hospital, Taiwan from May 2018 to March 2019. All rabbits received a physical examination and underwent routine laboratory tests including CBCs, biochemistry, and complete urinalysis. Measurements of UPC ratio and uGGT index were also performed. Rabbits that met the inclusion criteria (Yeh et al., 2019) were classified as healthy. On the other hand, rabbits were classified as suspected KD group when one or more of the following abnormalities were found: (1) renal azotemia (CRE concentration exceeds 2.7 mg/dL) with non-concentrating urine (USG < 1.019), (2) persistent renal azotemia (>3 mo), (3) persistent renal proteinuria (UPC ratio > 0.47 for more than 3 mo), (4) abnormal findings on radiographic renal images such as renal cortical calcification, nephroliths in the renal pelvis or stone in ureter in lateral or ventrodorsally abdominal view, and/or (5) abnormal ultrasonographic renal images; which were defined by the discovery of a smaller kidney size (as reported by Banzato et al.; Banzato et al., 2015), the kidney height < 1.23 cm, width < 1.27 cm or length < 2.09 cm in parasagittal scanning plane) and hyperechoic change in the renal parenchymal or pelvic region. Therefore, animals were divided into two experimental groups (healthy and KD group) based on clinical criteria indicated above.

### Sample Collection and Processing

Blood samples were obtained from the external saphenous vein using a 26-gauge needle (Becton, Dickinson and Company, USA). One mL of plasma (K2-EDTA) was obtained for hematological analysis. For biochemical analysis, 250 microliters of blood containing either no additive (Darwin Animal Hospital) or Lithium Heparin (Becton, Dickinson and Company, USA) (National Animal Hospital and National Taiwan University Veterinary Teaching Hospital) were centrifuged at 6,708 *g* (Eppendorf Centrifuge MiniSpin) for 2 min and the supernatant was collected for further analysis.

### Analytes

Erythrocytic indices were obtained by the Exigo Veterinary Hematology Analyzer (Boule Diagnostics AB, Sweden) at Darwin Animal Hospital, and the ProCyte DX analyzer (IDEXX Laboratories, USA) at National Animal Hospital and National Taiwan University Veterinary Teaching Hospital. The following hematological parameters were measured: hematocrit (HCT), hemoglobin (HGB), red blood cell count (RBC), mean cell volume (MCV), mean corpuscle hemoglobin (MCH), MCH concentration (MCHC), red cell distribution width (RDW); total white blood cell count (WBC), and platelet (PLT) count, mean platelet volume.

The biochemical values were analyzed by SPOTCHEM™ (EZ SP-4430, ARKRAY, Inc., Japan) automated dry biochemical analyzer and IDEXX Catalyst (IDEXX Laboratories, USA). The following parameters were measured: total protein, albumin (ALB), globulin (GLOB), blood urea nitrogen (BUN) and creatinine (CRE), calcium (CA), phosphate (P), sodium (NA), potassium (K) and chloride (CL). The method used for BUN detection is a colorimetric reaction using o‐phthaldehyde/N‐1‐ naphthyl‐N0‐diethylethylenediamine, and 3,5‐dinitrobenzoic acid for CRE.

Urine samples were collected by the free-catch method. The physical characteristics were recorded, and the chemical constituents were analyzed with either Combur-Test® Strips (Roche Diagnostics, Switzerland.), IDEXX UA™ Strips (IDEXX Laboratories, USA), or Arkray Test Strips (ARKRAY, Inc., Japan). The dipstick reactions were read by a Cobas U411 Urine Analyzer (Roche Diagnostics, Switzerland.), IDEXX VetLab® UA™ Analyzer (IDEXX Laboratories, USA), and PocketChem UA PU-4010 (ARKRAY, Inc., Japan) respectively to avoid variability resulting from visual inspection. The urine sediments were examined with microscopy, and samples with active urine sediment were excluded from the analysis to preliminarily rule out the possibility of urinary tract infection. Centrifugation of the urine samples was performed (H-18, Kokusan Co. Ltd., Japan) at 3,500 rpm for 15 min to collect supernatants for urinary indices analysis. The urine protein concentrations were determined by the reference assay (pyrogallol red-molybdate complex method), urine creatinine (uCRE) concentration was measured with the same assay as serum CRE (modified Jaffe test), and urine GGT (uGGT) was measured with the same assay as serum GGT (Szazs method) on the Beckman Coulter AU480 wet chemistry/photometric system (Beckman Coulter Inc., USA). The UPC ratio was calculated by dividing the urine protein concentration by the uCRE concentration; the GGT index by dividing the uGGT concentration by the uCRE concentration. The USG was measured by a refractometer (Atago, Japan). The urinary system of rabbits was evaluated with the Mindray 2200Vet Ultrasound system (Mindray Bio-Medical Electronics Co., Ltd., China) for kidney abnormalities.

### Statistical Analysis

Identification of outliers and determination of RIs for urinary indices were performed following the ASVCP guidelines using Reference Value Advisor 2.1 (Microsoft Corp., USA; Geffré et al., 2011) and guidelines as described in our previous study (Yeh et al., 2019). Urinary results from healthy pet rabbits were compared with two studies: source 1: Reusch et al., 2009; and source 2: Mancinelli et al., 2012. Table 1 outlines the comprehensive reference sources and the characteristics of their study population.

**Table 1.** Reference RIs for comparison of population characteristics in the current study

Reference source		Rabbit species	Age	Sex	Sample size
1	Reusch et al., 2009	Domestic rabbits (*Oryctolagus cuniculus*), breeds including Fancy (2 Belgian hare, 1 Continental giant, 15 Dwarf lop, 25 French lop, 2 Lionhead), Fur (3 Satin, 2 Rex), and 4 Crossbreeds. 38 of the rabbits were used for breeding and 16 were pets.	Ranged from 4 mo to 7 yrs	21 M (14E, 7N); 33 F (28E, 5N)	54
2	γMancinelli et al., 2012	Domestic rabbits (*Oryctolagus cuniculus*), breeds including dwarf lop (*n* = 19), crossbreed (*n* = 7), Netherland dwarf, (*n* = 5), English cross lop (*n* = 3), lion-head (*n* = 2), lion-head cross lop (*n* = 2), French lop (*n* = 2), and chinchilla (*n* = 1).	Ranged from 3 mo to 11 yrs	15 M (6E, 9N); 26 F (13E, 13N)	41

M, Male; F, Female; E, Entire; N, Neutered.

The normality of each data was tested with the Shapiro–Wilk test. To assess the difference between groups, Student’s *t*-test was utilized for Gaussian distributed data and represented as the mean with standard deviation; the Mann–Whitney *U*-test was used for non-Gaussian distributed data and represented as the median with interquartile range (IQR). The differences in categorical parameters were analyzed using Chi-square/Fisher’s exact test where

## Results

### Study Population

Thirty healthy and 11 KD crossbred pet rabbits were enrolled in the study. Table 2 shows the characteristics of the study population. Among these 11 KD patients, 6 rabbits showed abnormal renal ultrasonography. Sex and body weight did not differ between groups. Rabbits with KD were significantly older than the healthy control group. Among 11 rabbits with KD, 63% showed 5% to 10% dehydrated and 63% were underweight with a body condition score of 1 or 2 out of 5.

**Table 2.** Characteristics of the study population

Parameters	Healthy group (*n* = 30)	KD group (*n* = 11)	*P* value
Sex (female:male)^a^	21:9	6:5	0.355
Age (mo)^b^	17 (29)	89 (30)	<0.001*
BW, kg^c^	1.60 (0.07)	1.55 (0.13)	0.732
BCS (1/5:2/5:3/5:4/5:5/5)^a^	0:0:11:19:0	2:5:3:1:0	<0.001*
Hydration (< 5%:5% to 10%)^a^	30:0	4:7	<0.001*

a: Chi-square test: represented as the sample number; b: Kruskal–Wallis test: represented as median (interquartile range; IQR); c: Student’s *t*-test: represented as mean (standard deviation, SD).

### Urinary Indices in Healthy Crossbred Pet Rabbits (*n* = 30) and the Comparison to Two References

Table 3 describes the descriptive statistics and the RIs of urinary indices in healthy cross-bred pet rabbits. Among the 30 healthy pet rabbits, 100% showed urine protein levels below the lower reference limit reported in reference source 1. Additionally, 20% of healthy rabbits had an abnormal UPC ratio: 14% (4/30) were below the lower reference limit, while 6.7% (2/30) were above the upper reference limit. All rabbits had normal USG, based on reference source 1. Only one rabbit had an abnormal uGGT and uGGT index above the upper reference limit, based on reference source 2.

**Table 3.** Descriptive statistics and reference intervals of urinary indices in healthy cross-breed pet rabbits (*n* = 30)

Analytes	Descriptive statics				Reference interval		D^*^	M^‡^	O^†^
	N	Mean ± SD	Median	Range	LRL (90% CI)	URL (90% CI)			
uPro, mg/dL	30	27.7 ± 9.7	28.0	7.0 to 46.0	7.5 (2.9 to 12.5)	47.8 (42.6 to 52.8)	G	P	0
uCRE, mg/dL	30	147.7 ± 81.0	137.5	25 to 350	20.7 (29.4 to 64.6)	316.1 (268.1 to 358.1)	G	P	0
UPC ratio	30	0.21 ± 0.09	0.19	0.09 to 0.44	0.074 (0.060 to 0.095)	0.453 (0.365 to 0.556)	G	P	0
uGGT, U/L	30	41.7 ± 24.6	38.0	7.0 to 120.0	7.2 (4.7 to 12.2)	105.8 (84.0 to 132.1)	G	PT	1(S)
uGGT index	30	0.31 ± 0.19	0.28	0.11 to 1.07	0.09 (0.07 to 0.12)	1.01 (0.67 to 1.59)	G	PT	0
USG	30	1.036 ± 0.009	1.035	1.020 to 1.050	1.017 (1.011 to 1.022)	1.055 (1.050 to 1.060)	G	P	0

N indicates number of animals; URL, upper reference limit; LRL, lower reference limit; CI, confidence interval. ^*^D, Distribution; G, Gaussian; NG, non- Gaussian. ^‡^M, Method: P, parametric; PT, transformed, data transformed to Gaussian distribution prior to applying parametric methods; NP, nonparametric. ^†^O, Outliers; (S) indicates that suspect outliers were present and included; (R) indicates that discrepant outliers were present and removed.

### Clinicopathological Variables in Healthy and KD Rabbits

**Figure 1.**
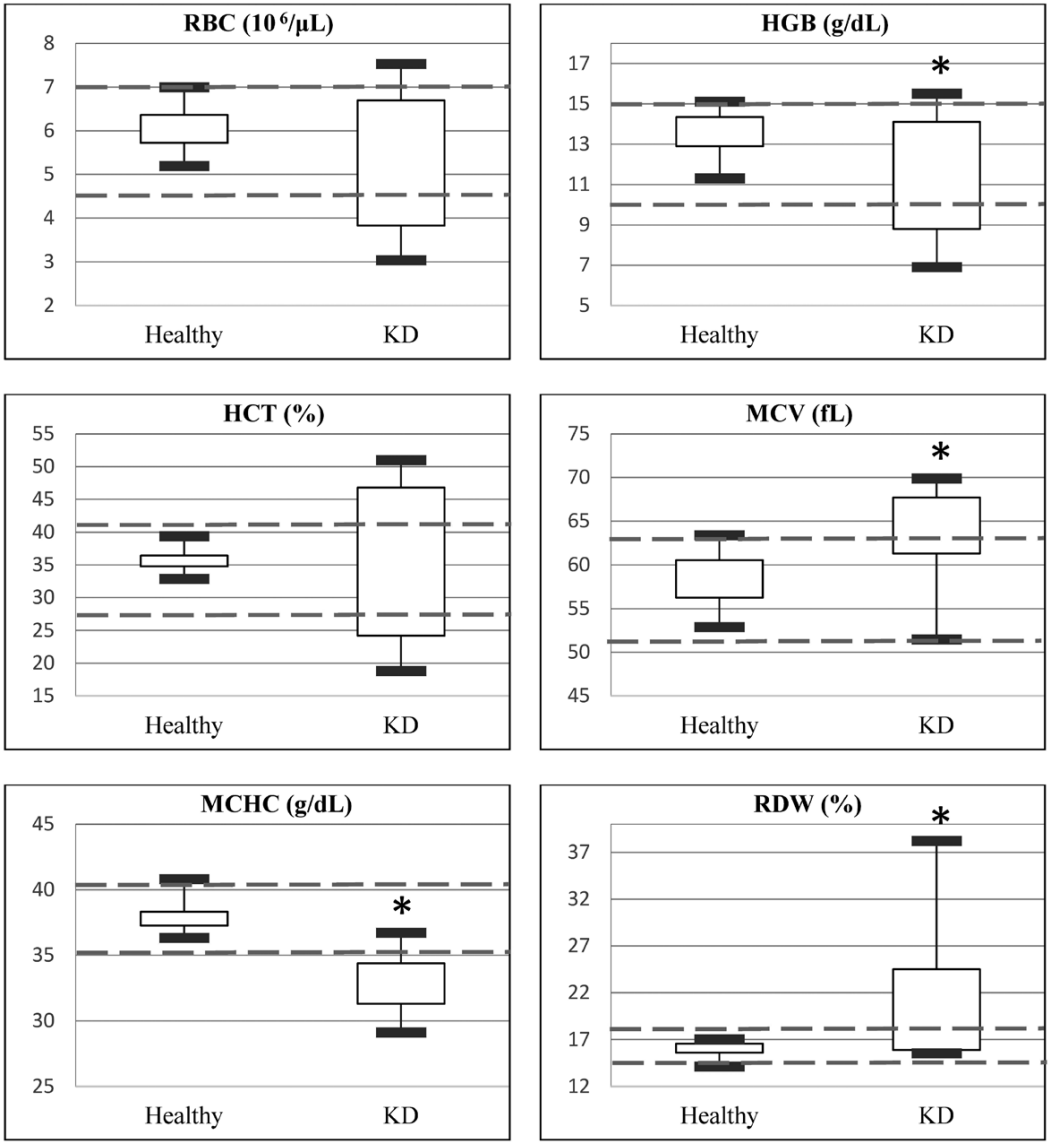
Box and whisker plot for erythrocytic indices that exhibited both significant differences and clinical relevance between the healthy pet rabbits and those with kidneydisease (KD). The central box represents the values from the lower to upper quartile (25 to 75 percentiles). The vertical line shows the minimum to the maximum value. The dashed lines represent the reference interval for those indices. *Statistically significant difference (*P* < 0.05).

**Figure 2.**
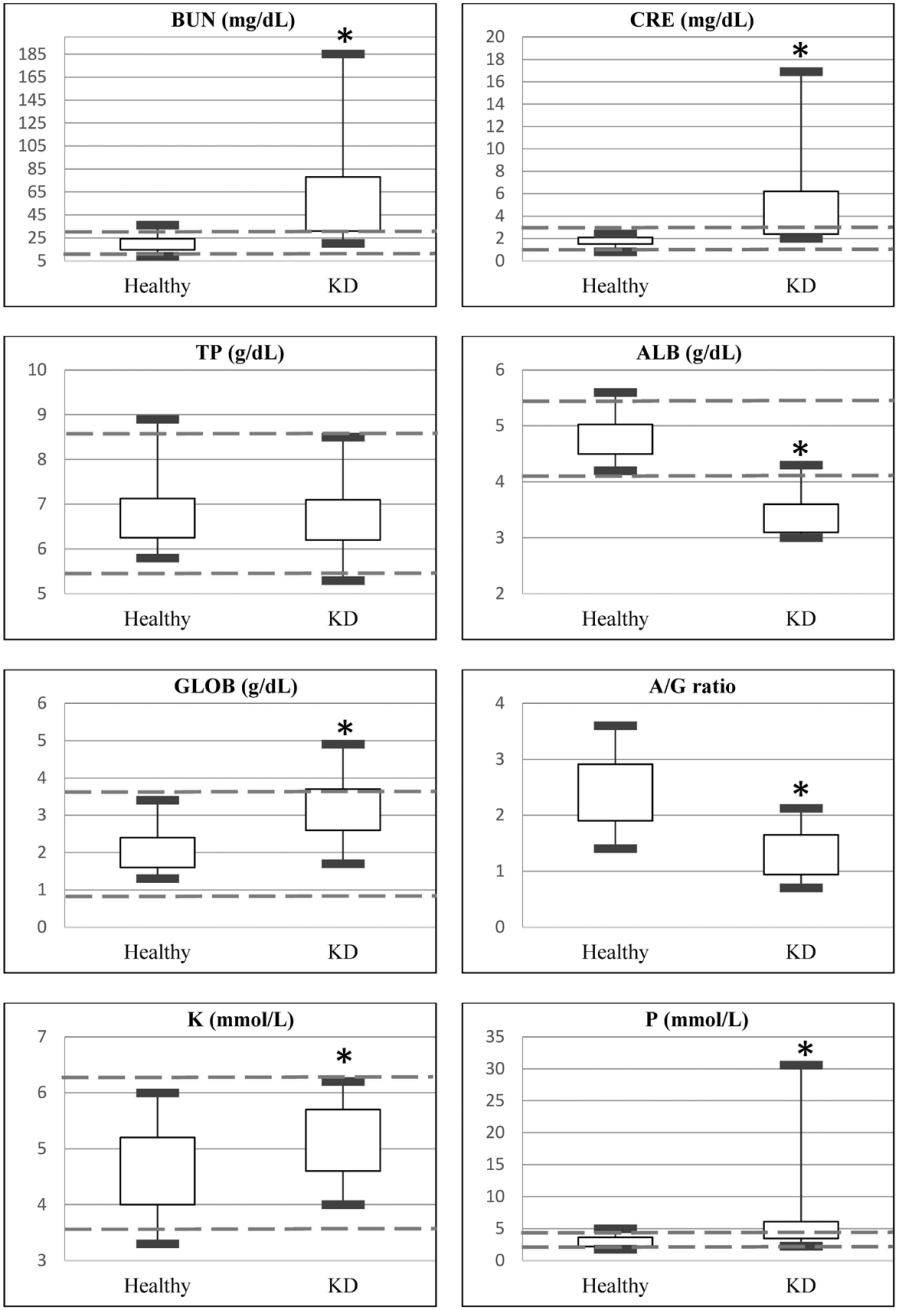
Box and whisker plot for biochemical indices that exhibited both significant differences and clinical relevance between the healthy pet rabbits and those with kidney disease (KD). The central box represents the values from the lower to upper quartile (25 to 75 percentiles). The vertical line shows the minimum to the maximum value. The dashed lines represent the reference interval for those indices. *Statistically significant difference (*P* < 0.05).

**Figure 3.**
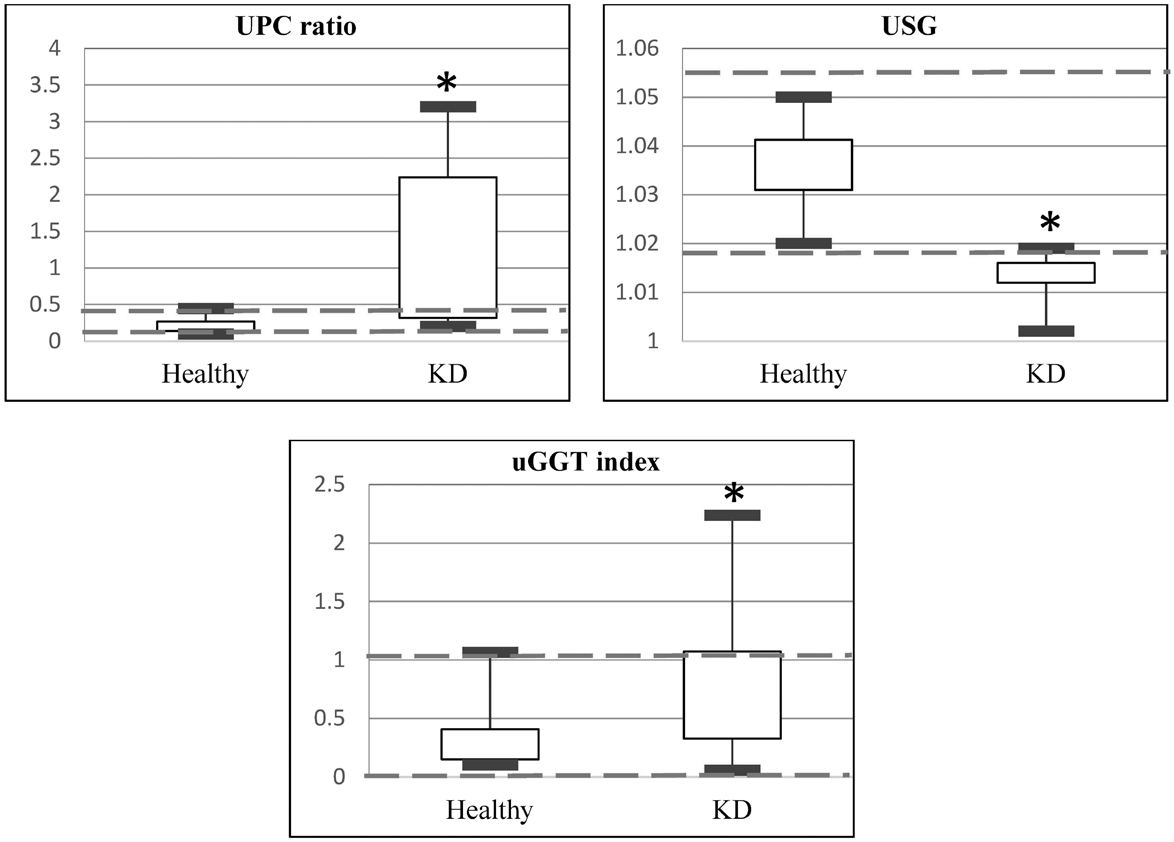
Box and whisker plot for urine indices that exhibited both significant differences and clinical relevance between the healthy pet rabbits and those with kidney disease (KD). The central box represents the values from the lower to upper quartile (25 to 75 percentiles). The vertical line shows the minimum to the maximum value. The dashed lines represent the reference interval for those indices. *Statistically significant difference (*P* < 0.05).

The clinicopathological results of both groups are shown in Table 4. Among the hematological results (Figure 1), rabbits with suspected KD had significantly lower HGB, MCH, and MCHC, and higher MCV, PLT, RDWa (average RDW), and RDW% compared to the healthy control group. Among the biochemistry results (Figure 2), rabbits with suspected KD had significantly higher BUN, CRE, GLOB, P, and K, and lower ALB and A/G ratio, compared to the healthy control group. For urinalysis (Figure 3), rabbits with suspected KD had significantly higher UPC ratio and uGGT index, whereas lower levels of uCRE and USG. Among categorical variables, urine color, urine turbidity, dipstick urine pH, and dipstick urine blood (occult blood) showed significant differences (*P* < 0.05) between the two groups.

**Table 4.** Clinicopathological parameters of the healthy cross-bred pet rabbits (*n* = 30) and rabbits with suspected kidney disease (KD, *n* = 11)

Parameters	Healthy group (N = 30)	KD group (*N* = 11)	*P* value	RI^‡^ (*N* = 85)
Hematology				
RBC, 10^6^/uL^b^	6.0 (0.6)	5.4 (2.9)	0.147	4.5 to 7.0
HGB, g/dL^b^	13.5 (1.5)	10.2 (5.3)	0.012*	10.1 to 15.0
HCT (%)^b^	35.5 (1.7)	31.7 (22.6)	0.139	27.5 to 41.3
MCV, fl^c^	59.1 (4.3)	66.0 (6.4)^†^	<0.001*	52.6 to 63.1
MCH, pg^b^	22.2 (1.0)	20.9 (1.9)	0.031*	20.1 to 23.7
MCHC, g/dL^b^	37.7 (1.0)	33.0 (3.1)^†^	<0.001*	35.7 to 40.6
PLT, 10^3^/uL^b^	218 (74)	313 (349)	0.011*	81.3 to 380.7
RDWa, fl^b^	42.3 (3.4)	46.8 (15.4)	0.001*	37.7 to 47.4
RDW, %^b^	16.1 (1.0)	18.3 (8.6)^†^	0.010*	14.9 to 17.7
MPV, %^c^	5.9 (0.1)	6.2 (0.2)	0.223	5.2 to 7.0
WBC, /uL^c^	5400 (284)	5588 (723)	0.315	3500 to 1020
Biochemistry				
BUN, mg/dL^b^	18 (10)	56 (47)^†^	<0.001*	10.1 to 31.1
CRE, mg/dL^b^	1.9 (0.5)	3.0 (3.8)^†^	<0.001*	1.1 to 2.7
TP, g/dL^c^	6.7 (0.1)	6.6 (0.3)	0.747	5.5 to 8.6
ALB, g/dL^b^	4.8 (0.5)	3.5 (0.5)^†^	<0.001*	4.1 to 5.5
GLOB, g/dL^c^	1.9 (0.1)	3.1 (0.3)	0.001*	0.9 to 3.6
A/G ratio^b^	2.6 (1.3)	1.0 (0.7)	<0.001*	—
P, mg/dL^b^	3.0 (1.4)	4.6 (2.6)^†^	0.016*	1.9 to 4.3
NA, mmol/L^b^	142 (6)	142 (5)	0.653	126 to 152
K, mmol/L^b^	4.7 (1.2)	5.5 (1.3)	0.012*	3.7 to 6.5
CL, mmol/L^b^	111 (9)	110 (17)	0.965	101.6 to 131.1
Urinalysis				
uPro, mg/dL^b^	28 (13)	49 (55)^†^	0.075	7.5 to 47.8
uCRE, mg/dL^b^	137.5 (104)	55 (45)	<0.001*	20.7 to 316.1
uGGT, U/L^b^	38 (28)	35 (80)	1.000	7.2 to 105.8
UPC ratio^b^	0.19 (0.13)	0.77 (1.92)^†^	<0.001*	0.018 to 0.3999
uGGT index^b^	0.28 (0.26)	0.60 (1.03)	0.025*	0.09 to 1.01
USG^b^	1.035 (0.010)	1.014 (0.006)^†^	<0.001*	1.017 to 1.055
Urine color (Yellow: Amber: Brown: Orange)^a^	2:13:14:1	8:3:0:0	0.003*	
Urine turbidity (Clear: Turbid)^a^	1:29	11:0	<0.001*	
dPRO (0+: to /+: 1+: 2+: 3+)^a^	19:3:6:1:1	5:1:4:1:0	0.691	
dPH (5: 7: 8: 9) ^a^	0:2:9:19	3:0:5:3	0.027*	
dBLD (0+: −/+: 1+: 2+: 4+)^a^	27:1:1:1:0	5:0:3:1:2	0.011*	
dKET (0+: −/+: 1+)^a^	19:2:9	11:0:0	0.064	
dBIL (0+: 1+: 2+)^a^	27:2:1	11:0:0	0.552	
dGLU (0+: −/+: 1+: 2+)^a^	27:1:1:1	7:0:2:2	0.117	

a: Chi-square test: represented as sample number; b: Kruskal–wallis test: represented as median (interquartile range; IQR); c: Student’s t-test: represented as mean (standard deviation, SD). Dipstick results: protein (dPRO), blood (dBLD), ketone (dKET), bilirubin (dBIL), glucose (dGLU). ^‡^RI from Yeh et al., 2019 [7]. ^†^Values < Lower Reference Limit or > Upper Reference Limit of the compared RI. ^*^Statistically significant difference (*P* < 0.05). The > 5% dehydration status was defined as increased skin tenting and dry oral mucous membranes.

## Discussions

Our study demonstrates the potential of urinary parameters—specifically USG, UPC ratio, and uGGT index—as indicators for KD in rabbits. While these biomarkers are well-established in dogs and cats, their application in pet rabbits remains underexplored and underutilized in clinical practice. The present study described the urinary parameters specifically in the crossbred pet rabbit population.

Comparison with existing literature highlights, notable differences were found in urine protein levels between our crossbred study population and predominantly pure-breed reference source 1. Reference source 1 established the RIs mainly from large-sized (1.4 to 7.6 kg) pure-breed rabbits that were predominantly (70%) raised for breeding or show purposes. Reference source 2 provides RIs for uGGT and the uGGT index, with a population sample of small- to medium-sized pure-breed rabbits weighing between 0.6 and 7 kg. In contrast, the rabbits from the present study weighed between 0.89 and 2.9 kg and were entirely composed of crossbred pet rabbits. Comparing our findings with these references revealed notable differences, particularly in urine protein levels, which were significantly lower in our crossbred population. Indeed, different equipment and methodologies can also contribute to variations in laboratory results, potentially affecting the consistency and accuracy of urinary indices. Factors such as the sensitivity, specificity, and calibration of the instruments used can introduce variability. For instance, differences in the detection limits of analyzers or variations in the reagents employed might lead to discrepancies in the measured values of these urinary markers. However, if the entire study population consistently falls outside the established RIs, it is unlikely that these variations are solely due to technical differences. This discrepancy may be attributed to breed-associated variation, as our study involved smaller, crossbred rabbits. Similar correlations between body mass and urine protein have been observed in human medicine, where body mass index is associated with total urine protein and albumin excretion (Toto et al., 2010), and obesity is a risk factor for proteinuria and end-stage renal disease (Iseki et al., 2004). Research reports with similar RIs of urine protein to the present study could also be found in the literature, however, they are either obtained from unknown numbers of rabbits (0 to 3.3 g/L) (Oglesbee, 2011) or a study with a relatively small sample size (<0.5 g/L, *n* = 7 NZWs) (Easley and Halliwell, 1977). Notably, there were no significant discrepancies in USG and uGGT indices between our study and the published references. The consistency in these parameters suggests they may be less influenced by breed and body size variations. However, further research is needed to investigate any potential correlation between uGGT and body mass.

USG, reflective of renal tubule function in concentrating or diluting urine, is species-specific. So far, the RI for USG in rabbits is not well studied. Urinary GGT is an indicator of acute renal tubular injury. In our study, although a statistically significant difference in the uGGT index was found between groups, there was considerable overlap, indicating a single parameter was insufficient to identify possible renal dysfunction. The mild elevation of the uGGT index may be partially explained by the nature of the studied cases, which were mostly rabbits with suspected KDs that were stably controlled. However, the exact location of the injury remains unconfirmed. The physical characteristics of urine in the KD were clearer, more acidic, and more frequently positive for occult blood compared to the control group. Clear and acidic urine often indicates diluted urine associated with poor prognosis and is commonly seen in end-stage CKD when kidneys cease excreting calcium (Harcourt-Brown, 2013). Consistent with the literature, the higher UPC ratio observed in the KD underscores its relevance as a diagnostic indicator in rabbits (Selleri et al., 2015).

While urine dipsticks serve as a preliminary tool for detecting proteinuria in dogs and cats, their efficacy in rabbits remains limited due to weak correlations with quantitative urine protein levels and UPC ratios observed in our study. A recent study suggested that when USG is ≤ 1.024, the dipstick can have a good positive predictive value for detecting proteinuria (UPC > 0.3) in rabbits, while the overall negative predictive values are poor (Agúndez and Porquet, 2021).

Although not the primary focus of this study, we discuss the well-established physical, hematological, and biochemical characteristics of crossbred pet rabbits with KD compared to healthy individuals, as data in this breed are limited. Clinical signs in KD rabbits include older age, lower body condition scores, and dehydration, consistent with literature describing chronic KD as a condition affecting older animals (Bartges, 2012). Significant weight loss is typical in CKD rabbits, despite maintained appetite, often resulting in a 30% to 40% decrease in body weight and cachexia (Eddy et al., 1986; Tvedegaard, 1987).

Among erythrocytic indices, KD rabbits showed lower but still within normal range levels of RBC, HGB, and HCT, suggesting potential subclinical anemia. Increased RDW (%) in the KD group indicates anisocytosis, associated with declining glomerular filtration rate and chronic inflammation, affecting RBC maturation (Weiss and Goodnough, 2005; Lippi et al., 2008). Notably, there was no significant difference in WBC counts between groups; leukocytosis is not a reliable marker of inflammation in rabbits (Toth and Krueger, 1988, 1989). Alterations in the neutrophil-to-lymphocyte ratio may better indicate infection (Melillo, 2007).

For biochemistry results, KD rabbits presented azotemia, hypoalbuminemia, hyperphosphatemia, and elevated GLOB, albeit with normal mean/median values. Hypoalbuminemia and a decreased A/G ratio were likely attributed to protein-losing nephropathies, advanced hepatic disease, or chronic malnutrition, as observed in rabbit studies (Melillo, 2007). Hyperphosphatemia, along with azotemia and hypercalcemia, is indicative of a poor prognosis, as reported in equine research (Schott, 2007). Conversely, although not observed in this study, hypophosphatemia might occur during the early stages of CKD due to reduced intestinal absorption, as documented in canine and feline studies (Vaden, 2011).

Electrolyte analysis revealed higher potassium levels in the KD group, despite being within the normal range, influenced by tubular and glomerular damage causing protein-losing nephropathy. Rabbits are susceptible to electrolyte imbalances due to their complex GI physiology. Normokalemia or hypokalemia may result from dietary insufficiency, alkalosis, or renal fluid loss in CKD (Harcourt-Brown, 2013). The study suggests that K regulation remains within normal limits but may be impacted by renal tubular and glomerular damage.

Rabbits are strictly herbivorous, with a natural diet consisting of herbage that is low in fiber but rich in protein and soluble carbohydrates. The occurrence and progression of renal disease in rabbits are significantly influenced by dietary components such as protein, phosphorus, calcium, lipids, and vitamin D (Klahr et al., 1983; Suckow et al., 2002). High-protein diets have been shown to affect renal concentrating capacity (Singer, 2003), whereas excessive phosphorus intake exacerbates nephrocalcinosis, particularly in the kidney cortex and medulla (Ritskes-Hoitinga et al., 2004). Diets high in calcium, such as those containing alfalfa meal, and excessive vitamin D intake, contribute to renal disease by inducing tissue calcification (Klahr et al., 1983; Ritskes-Hoitinga et al., 2004). While we did not record the diet history in the current study, the role of dietary composition in the development and management of renal disease in rabbits can not be overlooked.

This study has several limitations. Firstly, the small sample size in both groups is relatively small, and the type of KD within the study population was not further defined. However, our study addresses a critical gap by investigating urinary indices in crossbred pet rabbits, a population traditionally underrepresented in research. Secondly, variability in sample collection and clinicopathological data from different clinics, as well as the use of different equipment, could impact result consistency. However, when comparing the technical variations to disease-related changes, the effect size might be minimal.

## Conclusions

In conclusion, this study established RIs for urinary indices based on 30 clinically healthy purebred pet rabbits. Our findings emphasize the clinical importance of these urinary indices for detecting renal abnormalities in crossbred pet rabbits. Significant differences in basic urinary physical characteristics between healthy and KD groups were also identified, underscoring the clinical relevance of this quick and inexpensive test, which should not be neglected. Our data highlight breed-related variations, particularly in urine protein levels and UPC ratios, compared to existing literature. Furthermore, various clinicopathological abnormalities in rabbits with KD, beyond traditional markers such as BUN and creatinine, might provide valuable insights for veterinarians evaluating renal health in pet rabbits.

## References

[CIT0001] Agúndez, M. G., and N. C. Porquet. 2021. Evaluation of urine dipstick for proteinuria assessment in pet rabbits. Vet. Rec. 188:e306. doi: https://doi.org/10.1002/vetr.306 33870527

[CIT0002] Banzato, T., L. Bellini, B. Contiero, P. Selleri, and A. Zotti. 2015. Abdominal ultrasound features and reference values in 21 healthy rabbits. Vet. Rec. 176:101. doi: https://doi.org/10.1136/vr.102657 25362002

[CIT0003] Bartges, J. W. 2012. Chronic kidney disease in dogs and cats. Vet. Clin. North Am. Small Anim. Pract. 42:669–692. doi: https://doi.org/10.1016/j.cvsm.2012.04.008 22720808

[CIT0004] Easley, J. R., and W. H. Halliwell. 1977. Relationship of proteinuria to glomerular basement membrane deposits in serum-sickness glomerulonephritis in rabbits. Vet. Pathol. 14:482–489. doi: https://doi.org/10.1177/030098587701400508 919239

[CIT0005] Eddy, A. A., R. J. Falk, R. K. Sibley, and T. H. Hostetter. 1986. Subtotal nephrectomy in the rabbit: a model of chronic hypercalcemia, nephrolithiasis, and obstructive nephropathy. J. Lab. Clin. Med. 107:508–516. 3711720

[CIT0006] Geffré, A., D. Concordet, J. P. Braun, and C. Trumel. 2011. Reference Value Advisor: a new freeware set of macroinstructions to calculate reference intervals with Microsoft Excel. Vet. Clin. Pathol. 40:107–112. doi: https://doi.org/10.1111/j.1939-165X.2011.00287.x 21366659

[CIT0007] Harcourt-Brown, F. M. 2013. Diagnosis of renal disease in rabbits. Vet. Clin. North Am. Exot. Anim. Pract. 16:145–174. doi: https://doi.org/10.1016/j.cvex.2012.10.004 23347542

[CIT0008] Iseki, K., Y. Ikemiya, K. Kinjo, T. Inoue, C. Iseki, and S. Takishita. 2004. Body mass index and the risk of development of end-stage renal disease in a screened cohort. Kidney Int. 65:1870–1876. doi: https://doi.org/10.1111/j.1523-1755.2004.00582.x 15086929

[CIT0009] Klahr, S., J. Buerkert, and M. L. Purkerson. 1983. Role of dietary factors in the progression of chronic renal disease. Kidney Int. 24:579–587. doi: https://doi.org/10.1038/ki.1983.197 6363797

[CIT0010] Kraus, A. L., S. H. Weisbroth, R. E. Flatt, and N. Brewer. 1984. Biology and diseases of rabbit. In: Fox, J. G., B. J. Cohen, and F. M. Loew, eds., Laboratory animal medicine. 1st ed. Orlando (FL): Elsevier; p. 207–240. doi:doi: https://doi.org/10.1016/B978-012263951-7/50012-0

[CIT0011] Lippi, G., G. Targher, M. Montagnana, G. L. Salvagno, G. Zoppini, and G. C. Guidi. 2008. Relationship between red blood cell distribution width and kidney function tests in a large cohort of unselected outpatients. Scand. J. Clin. Lab. Invest. 68:745–748. doi: https://doi.org/10.1080/00365510802213550 18618369

[CIT0012] Mancinelli, E., D. J. Shaw, and A. L. Meredith. 2012. γ-Glutamyl-transferase (GGT) activity in the urine of clinically healthy domestic rabbits (*Oryctolagus cuniculus*). Vet. Rec. 171:475. doi: https://doi.org/10.1136/vr.101081 23092973

[CIT0013] Melillo, A. 2007. Rabbit clinical pathology. J. Exot. Pet Med. 16:135–145. doi: https://doi.org/10.1053/j.jepm.2007.06.002 32362792 PMC7185592

[CIT0014] Meredith, A., and P. A. Flecknell. 2006. BSAVA manual of rabbit medicine and surgery. 2nd ed. Gloucester (UK): British Small Animal Veterinary Association.

[CIT0015] Oglesbee, B. L. 2011. Blackwell’s five-minute veterinary consult: small mammal. 2nd ed. Ames (IA): Wiley-Blackwell.

[CIT0016] Percy, D. H., and S. W. Barthold. 2013. Pathology of laboratory rodents and rabbits. 3rd ed. Ames (IA): Blackwell Publishing.

[CIT0017] Reavill, D. R., and A. M. Lennox. 2020. Disease overview of the urinary tract in exotic companion mammals and tips on clinical management. Vet. Clin. North Am. Exot. Anim. Pract. 23:169–193. doi: https://doi.org/10.1016/j.cvex.2019.09.003 31759446 PMC7110585

[CIT0018] Reusch, B., J. K. Murray, K. Papasouliotis, and S. P. Redrobe. 2009. Urinary protein: creatinine ratio in rabbits in relation to their serological status to *Encephalitozoon cuniculi*. Vet. Rec. 164:293–295. doi: https://doi.org/10.1136/vr.164.10.293 19270319

[CIT0019] Ritskes-Hoitinga, J., H. N. A. Grooten, K. J. H. Wienk, M. Peters, A. G. Lemmens, and A. C. Beynen. 2004. Lowering dietary phosphorus concentrations reduces kidney calcification, but does not adversely affect growth, mineral metabolism, and bone development in growing rabbits. Br. J. Nutr. 91:367–376. doi: https://doi.org/10.1079/BJN20041065 15005822

[CIT0020] Schott, H. C. 2007. Chronic renal failure in horses. Vet. Clin. North Am. Equine Pract. 23:593–612. doi: https://doi.org/10.1016/j.cveq.2007.10.002 18061852

[CIT0021] Selleri, P., L. Bongiovanni, G. Isani, L. Della Salda, and N. Di Girolamo. 2015. Protein to creatinine ratio in pet rabbits with suspect or histologically confirmed renal disease. In: Proceedings Association of Exotic Mammal Veterinarians, 13th Annual Conference, Paris, France. p. 333.

[CIT0022] Shiga, T., M. Nakata, Y. Miwa, F. Kikuta, N. Sasaki, T. Morino, and H. Nakayama. 2022. Age at death and cause of death of pet rabbits *(Oryctolagus cuniculus)* seen at an exotic animal clinic in Tokyo, Japan: a retrospective study of 898 cases (2006–2020). J. Exot. Pet Med. 43:35–39. doi: https://doi.org/10.1053/j.jepm.2022.09.003

[CIT0023] Singer, M. A. 2003. Dietary protein-induced changes in excretory function: a general animal design feature. Comp. Biochem. Physiol. B: Biochem. Mol. Biol. 136:785–801. doi: https://doi.org/10.1016/j.cbpc.2003.08.012 14662303

[CIT0024] Suckow, M. A., D. W. Brammer, H. G. Rush, and C. E. Chrisp. 2002. Biology and diseases of rabbits. Chap. 9. 2nd ed. Laboratory Animal Medicine, American College of Laboratory Animal Medicine; Academic Press, Cambridge, Massachusetts. p. 335–336.

[CIT0025] Toth, L. A., and J. M. Krueger. 1988. Alteration of sleep in rabbits by *Staphylococcus aureus* infection. Infect. Immun. 56:1785–1791. doi: https://doi.org/10.1128/iai.56.7.1785-1791.1988 3384477 PMC259478

[CIT0026] Toth, L. A., and J. M. Krueger. 1989. Hematologic effects of exposure to three infective agents in rabbits. J. Am. Vet. Med. Assoc. 195:981–986. 2529233

[CIT0027] Toto, R. D., T. Greene, L. A. Hebert, L. Hiremath, J. P. Lea, J. B. Lewis, V. Pogue, M. Sika, and X. Wang. 2010. Relationship between body mass index and proteinuria in hypertensive nephrosclerosis: results from the African American Study of Kidney Disease and Hypertension (AASK) cohort. Am. J. Kidney Dis. 56:896–906. doi: https://doi.org/10.1053/j.ajkd.2010.05.016 20801567 PMC4517588

[CIT0028] Tvedegaard, E. 1987. Arterial disease in chronic renal failure--an experimental study in the rabbit. Acta Pathol. Microbiol. Immunol. Scand. A. 290:1–28. 3477940

[CIT0029] Vaden, S. L. 2011. Glomerular disease. Top. Companion Anim. Med. 26:128–134. doi: https://doi.org/10.1053/j.tcam.2011.04.003 21782143

[CIT0030] Weiss, G., and L. T. Goodnough. 2005. Anemia of chronic disease. N. Engl. J. Med. 352:1011–1023. doi: https://doi.org/10.1056/NEJMra041809 15758012

[CIT0031] Yeh, S. Y., C. H. Sung, T. L. Lin, T. L. Cheng, and C. C. Chou. 2019. The effects of crossbreeding, age, and sex on erythrocyte indices and biochemical variables in crossbred pet rabbits (*Oryctolagus cuniculus*). Vet. Clin. Pathol. 48:469–480. doi: https://doi.org/10.1111/vcp.12775 31556159

